# A common DNA deletion altering the 3’UTR of *mdr1* is associated with reduced mefloquine susceptibility in *P. vivax* parasites from Cambodian patients

**DOI:** 10.21203/rs.3.rs-6889263/v1

**Published:** 2025-09-01

**Authors:** Katie Ko, Kieran Tebben, Tsarasoa Andrianinarivomanana, Agnes Orban, Janne Grünebast, Virak Eng, Rotha Eam, Nimol Khim, Lionel Feufack-Donfack, Jeremy Salvador, Juliana Sá, Thomas Wellems, Dysoley Lek, Jean Popovici, David Serre

**Affiliations:** University of Maryland, Baltimore; University of Maryland, Baltimore; Institut Pasteur du Cambodge; Institut Pasteur du Cambodge; University of Maryland, Baltimore; Institut Pasteur du Cambodge; Institut Pasteur du Cambodge; Institut Pasteur du Cambodge; Institut Pasteur du Cambodge; Institut Pasteur du Cambodge; National Institute of Allergy and Infectious Diseases; National Institute of Allergy and Infectious Diseases; Centre for Parasitology, Entomology and Malaria Control,; Institut Pasteur du Cambodge; University of Maryland, Baltimore

## Abstract

Artemisinin-combination therapies (ACTs) are now recommended for the treatment of uncomplicated malaria caused by *Plasmodium vivax*, the parasite responsible for the majority of malaria infections outside of Africa. We analyzed the genome sequences of 206 *P. vivax* parasites collected from Cambodian malaria patients and showed that more than 80% of them carried a DNA deletion located immediately downstream of the multidrug resistance 1 gene (*mdr1*). This 837 bp deletion overlapped with a different deletion present at low frequency in South American isolates, suggesting a functional role despite not altering the coding sequence of *mdr1*. Using RNA sequencing, we showed that these deletions altered the transcripts expressed from *mdr1* and resulted in mRNAs with different 3’ untranslated regions. In Cambodian isolates, the deletion was significantly associated with a higher expression of *mdr1* and a lower *ex vivo* susceptibility to mefloquine. Finally, we genotyped 592 Cambodian isolates collected between 2014 and 2024 and showed that the *mdr1* deletion increased in frequency in Cambodia since the introduction of mefloquine as ACT partner drug. Overall, these findings indicate that a common deletion of a non-coding sequence affects the transcription, stability, or translation of *mdr1* in *P. vivax* parasites and could mediate reduced susceptibility to antimalarial drug(s) currently used for the treatment of uncomplicated vivax malaria.

## Introduction

Malaria remains a major public health problem that threatens half of the world’s population [1]. The disease is caused by infections with unicellular parasites of the *Plasmodium* genus, including *P. falciparum* that causes most of the deaths associated with malaria, and *P. vivax* that is responsible for the majority of cases outside of Africa [2]. *P. falciparum* and *P. vivax* are often co-endemic and malaria infections are frequently treated indiscriminately. While global efforts have led to substantial reductions in the burden of malaria caused by these two species, the unique biological features of *P. vivax* continue to hamper its control relative to the progress made against *P. falciparum*. These features include the ability of *P. vivax* stages to persist in dormant liver stages, which can reactivate months after a treatment that clears the blood infection, causing relapse infections. *P. vivax*, because of its preference for reticulocytes, also often circulates at low levels in the bloodstream, making its detection and diagnosis more challenging than *P. falciparum*. Finally, the lack of robust *in vitro* culture system for *P. vivax* complicates many studies of this parasite, including the assessment of antimalarial drug resistance [3] that often depends solely on patient studies and can be confounded by relapses and reinfections [4, 5].

Identification and validation of robust molecular markers for rapid and cost-effective surveillance of drug resistance is essential for improving malaria control and elimination efforts but has remained relatively unsuccessful for *P. vivax*, especially when compared to the progress in identifying *P. falciparum* drug resistance markers. Since the K76T mutation of the chloroquine resistance transporter (*crt*) gene is linked to chloroquine resistance in *P. falciparum* parasites, several studies have assessed its ortholog for a similar role in *P. vivax*. However, single nucleotide polymorphisms in *crt* have not associated with chloroquine resistance in field studies, nor in non-human primate models of *P. vivax* malaria [6].

Furthermore, engineered expression of *P. vivax crt* K76 or T76 polymorphisms showed no significant difference on their reductions of chloroquine accumulation in a *Dictyostelium* expression system [7]. On the other hand, some studies have suggested that increased expression of the crt gene, independent of specific point mutations, may have a role in mediating *P. vivax* chloroquine resistance. For example, a laboratory genetic cross in non-human primates showed linkage between increased *crt* transcription and an inherited chloroquine resistance phenotype [8]. Taken together, these findings suggest that gene regulation affecting the level of *crt* expression may be involved in the chloroquine responses of *P. vivax* malaria. Polymorphisms in the *P. vivax* multidrug resistance 1 (*mdr1*) gene, another important marker of antimalarial drug resistance in *P. falciparum*, have also extensively been examined for associations with antimalarial drug responses. Some studies have reported associations between specific mutations in *mdr1*, such as Y976F or G698S, and reduced sensitivity of *P. vivax* to chloroquine in *ex vivo* assays [9, 10]. However, the exact role of *mdr1* mutations and the extent of their contribution to chloroquine resistance remains unclear and sometimes contradictory [3, 9, 11]. Thus, while specific mutations of *mdr1* may be involved in antimalarial drug responses, their effects might vary depending on the genetic background of *P. vivax* strains or other unknown factors. Finally, DNA duplications of the *mdr1* gene have been suggested to influence antimalarial drug susceptibility [12, 13], similarly to findings in *P. falciparum* [14–16].

Due to increasing reports of potential *P. vivax* resistance to chloroquine [17–20], the first-line treatment for the blood stages of *P. vivax* since the 1950s, and the lack of robust molecular markers of resistance, the WHO now recommends using ACTs for treating uncomplicated vivax malaria worldwide, to both ensure efficacious clearance of blood stage P. vivax infections and to limit the emergence of drug resistance (although chloroquine is also recommended in areas without indication of chloroquine resistance) [21]. In Cambodia, for example, the standard of care for uncomplicated malaria (caused by any species) was switched from chloroquine to dihydroartemisinin-piperaquine in 2012 and then again to artesunate-mefloquine in 2016 (and was fully implemented in 2017).

We have recently reported that some Cambodian *P. vivax* parasites were cleared slowly after artesunate treatment [22], which may enable them to outlast the short half-life of artesunate in the blood, especially in cases of incomplete patient adherence to the multi-day regimen. While we did not observe treatment failure in this study, this slow clearance upon artesunate treatment could theoretically facilitate the acquisition of resistance to the partner drugs and complicate malaria control and elimination efforts in Southeast Asia. Here, we analyze parasite DNA and RNA extracted from the blood of Cambodian vivax malaria patients and describe a common deletion immediately downstream of *mdr1*. We examine its consequences on *mdr1* expression and on antimalarial drug susceptibility, as well as analyze 592 DNA samples collected between 2014 and 2024 to examine temporal changes in the frequency of this deletion in Cambodia.

## Methods

### Sample collection

Parasite DNA and RNA samples were collected from participants in an open-label clinical trial (NCT04706130) designed to evaluate the efficacy of two regimens of primaquine [23]. Eligible individuals seeking treatment for malaria in the Kampong Speu province (Western Cambodia) and diagnosed with *Plasmodium vivax* by rapid diagnostic test were offered to participate in the study. All participants had uncomplicated vivax malaria (confirmed by PCR), were 15 years or older, and had hemoglobin concentration greater than 8 g/dL. Pregnant and breastfeeding women were excluded from this study. All enrolled patients or their guardians provided written informed consent, and assent was obtained for all patients aged 15–18 years old. Ethical clearance was obtained by the National Ethics Committee for Health Research of Cambodia (158-NECHR, 06/29/2020) and the University of Maryland IRB (HP-00091095), and the study was overseen by the NIH Division of Microbiology and Infectious Diseases (Protocol 20 − 0010). At enrollment (i.e., before drug treatment), we collected a venous blood sample from each participant and immediately stored 50 μL in trizol for RNA analyses and processed the rest of the blood on a cellulose column to remove white blood cells before storage at −80°C for DNA analyses.

We also analyzed a biobank of DNA from 592 *P. vivax* infected individuals from the Ratanakiri, Mondolkiri and Kampong Speu provinces and collected between 2014 and 2024.

### Whole genome sequencing and identification of sequence rearrangements in Cambodian isolates

We extracted parasite DNA from 230 leukocyte-depleted blood samples using the DNeasy blood and tissue kit (Qiagen) kit and prepared whole genome sequencing libraries using the NEBNext^®^ Ultra^™^ II FS DNA Library Prep Kit for Illumina sequencing (NEB) according to the manufacturers’ instructions. We sequenced each library on an Illumina NovaSeq 6000 to generate 23–50 million paired-end reads of 100 bp per sample (**Supplemental Table 1**).

To identify sequence rearrangements throughout the *P. vivax* genome, we first used Hisat2 [24] to map all reads to the P01 reference genome (version 67) [25]. We then used custom scripts to parse the resulting bam files and counted, for each sample, the number of read pairs with alignments indicative of deletions or tandem duplications per 100 bp non-overlapping window of the P01 genome [13, 26]. Briefly, read pairs aligning in the correct orientation but distant from each other by at least 1,000 bp were considered indicative of deletions (SAM flags 161/97 with insert size > = 1,000 bp and SAM flags 81/145 with insert size <= −1,000 bp), while read pairs aligning in incorrect orientation (tail to tail) and distant from each other by at least 1,000 bp were considered indicative of tandem duplications (SAM flags 161/97 with insert size <= −1,000 bp and SAM flags 81/145 with insert size > = 1,000 bp). To determine if a given 100 bp window contained more reads indicative of a specific sequence rearrangement than expected by chance, we compared the number of such reads observed in one sample to the number expected under a Poisson distribution. All windows with a false discovery rate < 0.01 were considered significant. Note that this analytical pipeline enables detection of a sequence rearrangement even if only some of the clones present in one sample carry it.

**Figure 1:**
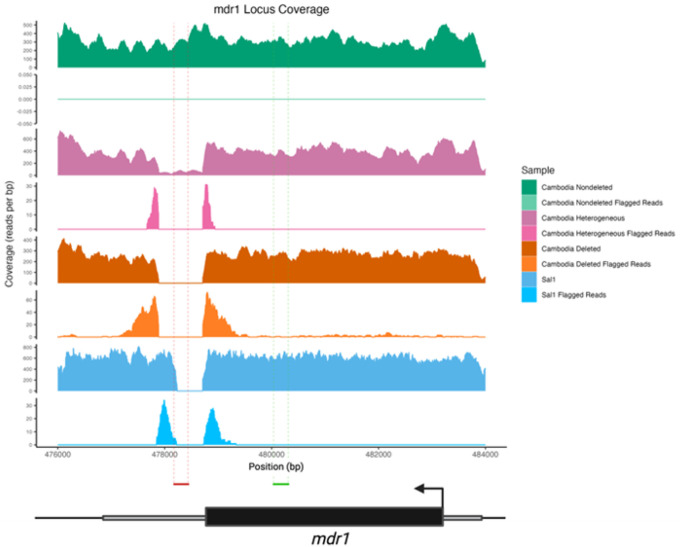
A common deletion downstream of the *mdr1* gene. Coverage plots of the *mdr1* locus for four *P. vivax* samples: (top to bottom) a Cambodian isolate in which all parasites carried the non-deleted sequence (green), a Cambodian isolate containing clones with and without the *mdr1* deletion (pink), a Cambodian sample in which all parasites contained the deletion (orange), and the NIH-1993-F3 clone derived from Salvador 1 which contains a different *mdr1* deletion (blue). For each sample, the upper row displays the overall read coverage relative to the P01 reference sequence, while the lower row displays the coverage of reads flagged for incorrect insert size and indicative of a deletion. The red horizontal bar indicates the region used to determine the number of reads mapped within the deletion, while the horizontal green bar indicates the unaffected region used for comparison.

We then evaluated, in samples where a *mdr1* deletion was detected, whether all or only some of the clones within one sample carried the *mdr1* deletion by comparing the number of reads mapped to a 400 bp portion within the *mdr1* deletion (between positions 478,100 and 478,500 of the P01 genome version 67) to the number of reads mapped to a 400 bp portion of the *mdr1* coding region that was not deleted or duplicated (between positions 480,000 and 480,400, Fig. 1). If the proportion of reads mapped to the deleted region represented less than 5% of the reads mapped to the control region, the sample was considered homogeneous for the deletion, while all other isolates were considered heterogeneous for the deletion (i.e., some clones carried the deletion and some clones carried the non-deleted sequence).

### Determination of the clonality of the Cambodian isolates

To estimate whether each isolate was monoclonal, we used GATK to calculate Fws [27] from the whole genome sequence data. We first masked regions of the genome containing multigene families and analyzed nucleotide positions successfully sequenced in at least 80% of samples and polymorphic in our dataset (only considering positions with two alleles). Samples with Fws > = 0.95 were considered monoclonal while samples with Fws < 0.95 were considered polyclonal.

### Nucleotide and amino acid sequence analyses

For each Cambodian *P. vivax* isolate deemed to be monoclonal, we used bcftools mpileup and custom scripts to reconstruct the nucleotide sequence of the *mdr1* protein-coding sequence using the gene coordinates from PlasmoDB. We then aligned these sequences in MEGA12 using MUSCLE [28] and generated Neighbor-Joining trees based on the number of differences between sequences, from both the nucleotide and translated amino acid sequences.

### Characterization of mdr1 sequence rearrangements in MalariaGEN samples

In order to characterize sequence rearrangements involving *mdr1* in a worldwide dataset, we analyzed 826 MalariaGEN PV4 *P. vivax* isolates from 25 countries in Africa, Asia, Oceania and America [29]. We remapped all sequences generated from these samples onto a 60 kb region surrounding the *mdr1* locus in the P01 reference genome (between positions 450,000 and 510,000 of chromosome 10) and screened these samples for deletions and tandem duplications by analyzing read pair orientation using the pipeline outlined above.

### Validation of mdr1 deletions by PCR amplification and Sanger sequencing

To confirm the *mdr1* deletions identified by whole genome analysis, we designed different sets of PCR primers to amplify the region surrounding the putative deletions downstream of *mdr1* (**Supplemental Table 2**). We amplified DNA from three Cambodian samples identified as homogenous for the deletion, as well as from NIH-1993-F3, a monkey-adapted *P. vivax* strain derived from the Salvador 1 strain that, based on sequence data previously generated [30], carries a deletion downstream of *mdr1* similar to those observed in South-American isolates from MalariaGEN. PCR amplification was performed using the Q5 high-fidelity master mix with an annealing temperature of 67°C and 35 cycles. The PCR products were loaded on a 1% agarose gel for 30 minutes and bands with the expected size were cut out, purified, and the DNA used for Sanger sequencing. The resulting DNA sequences were aligned to the P01 reference sequence to validate the deleted regions and their exact boundaries.

### RNA sequencing and gene expression analysis

We extracted RNA from 138 whole blood samples collected from the same infections as used in the whole genome sequencing analysis. Briefly, we used phenol/chloroform to extract RNA from trizol samples and, after ribosomal RNA depletion and polyA selection (NEB), we prepared RNA-seq libraries using the NEBNext Ultra II Directional RNA Library Prep Kit (NEB). We sequenced each library on an Illumina NovaSeq 6000 and generated ~ 30–414 million paired-end reads of 75 bp per sample. We aligned all reads using Hisat2 [24] to the *P. vivax* P01 genome with default parameters except for a maximal intron length set at 5,000 bp. PCR duplicates were removed from all files using custom scripts. Out of the 138 samples, 128 had more than 500,000 unique reads mapped to *P. vivax* protein-coding genes and were analyzed further (**Supplemental Table 3**).

We tested whether isolates carrying a *mdr1* deletion expressed mdr1 mRNA at a different level than isolates without deletion. To account for the transcript length difference caused by the deletion, we only counted reads mapped to the coding region of *mdr1* (that is unaffected by the deletion) and normalized this count by the total number of reads mapped to the *P. vivax* genome for each sample. We then used a t-test to compare the normalized *mdr1* expression of samples identified in our WGS analysis as being homogeneous for the deletion (n = 61) with those homogeneous for no deletion (n = 19).

### Association between mdr1 deletion and antimalarial drug susceptibility

We first compared the susceptibility to artesunate of *P. vivax* isolates carrying the *mdr1* deletion (n = 138) and isolates without the deletion (n = 29) based on genome sequence data and on PCR characterization of the presence/absence of the deletion. Artesunate susceptibility was expressed as the half-life of the parasite clearance slope determined *in vivo* from patients treated with artesunate [22, 23]. We also characterized by PCR the absence/presence of the *mdr1* deletion (**Supplemental Table 2**) in 14 *P. vivax* isolates for which the IC_50_ to mefloquine was determined *ex vivo* and tested whether these estimates were significantly associated with the deletion. *Ex vivo* mefloquine susceptibility assay was performed on freshly collected parasites using a ring-to-schizont maturation assay as previously described [31].

### Temporal changes in mdr1 deletion in Cambodian P. vivax

To measure changes in frequency of the *mdr1* deletion in the *P. vivax* Cambodian population over time, we screened by PCR DNA from 592 *P. vivax* isolates that were collected by the Institut Pasteur of Cambodia in the provinces of Ratanakiri, Mondolkiri and Kampong Speu between 2014 and 2024.

## Results

### A region immediately downstream of the mdr1 protein-coding sequence is frequently deleted in Cambodian P. vivax isolates

We analyzed the genomes of 206 Cambodian *P. vivax* isolates collected between 2021 and 2023 and sequenced at high coverage (> 30X, **Supplemental Table 1**) and screened them for deletions and tandem duplications. In 30 out of the 206 isolates (14.5%), we identified the duplication of the *dbp* gene (PVP01_0623800) that has been previously reported from Cambodian parasites [32]. Many other sequence rearrangements occurred only in a few samples but very few deletions and duplications were shared by many samples: in total, we only detected 26 deletions and 30 duplications present in more than 25% of the samples (**Supplemental Table 4**). These common sequence rearrangements typically involved genes belonging to multigene families, with 21 out of 26 deletions (81%) and 26 out of 30 duplications (87%) overlapping or neighboring genes annotated as PIR proteins, merozoite surface proteins (MSPs), serine-repeat antigens (SERAs), and tryptophan-rich proteins. The remaining sequence rearrangements involved *Plasmodium* exported proteins of unknown function, often PHIST proteins located in subtelomeric regions (**Supplemental Table 4**). The only sequence rearrangement frequently observed among the 206 Cambodian isolates that involved a single copy gene was a deletion located near the multidrug resistance 1 gene (*mdr1*, PVP01_1010900): 169 out of the 206 Cambodian *P. vivax* isolates (82%) carried a deletion of approximately 800 bp located immediately after the 3’ end of the *mdr1* protein-coding sequence (Fig. 1).

The analysis described above is only informative of the presence of a deletion: by only considering read pairs indicative of a deletion, this approach might overestimate the population frequency of the deletion if one sample contains clones both carrying the deletion and clones without. Since many *P. vivax* infections in Cambodia are polyclonal (e.g., [33, 34]), we evaluated whether some infections might be heterogeneous with regards to this deletion by comparing the sequence coverage in the deleted region with the coverage in a non-deleted section of the coding region of *mdr1* (Fig. 1). Of the 169 samples carrying a *mdr1* deletion, 146 (86%) were deemed homogenous for the deletion (i.e., all parasites carried the deletion, in orange in Fig. 1) and 23 (14%) heterogenous for the deletion (in pink in Fig. 1).

### The mdr1 deletion is present on different haplotypes

We then reconstructed the protein-coding sequence of *mdr1* using the genome sequence data generated from monoclonal isolates with or without the deletion. Surprisingly, we did not observe any clear clustering patterns separating samples with the deletion and those without using either the nucleotide sequence or the inferred amino acid sequence (**Supplemental Fig. 1**). In fact, we observed that the deletion was present on several distinct *mdr1* haplotypes that were also present in *P. vivax* parasites without the deletion (i.e., different sequences carried both the deleted and non-deleted sequences). This lack of linkage disequilibrium between the protein-coding sequence of *mdr1* and the downstream deletion suggests that either i) the deletion occurred independently several times on different genetic backgrounds (with, apparently, the exact same boundaries), or ii) that this deletion occurred some time ago and that recombination has re-shuffled it on different genetic backgrounds.

### Genomic analyses revealed the presence of two independent and overlapping deletions downstream of mdr1

To evaluate if the *mdr1* deletion observed in Cambodian *P. vivax* isolates was also present in other regions of the world, we re-analyzed 826 *P. vivax* samples that have been sequenced by the MalariaGEN project ([29], **Supplemental Table 5**). This dataset contains whole genome sequence data for *P. vivax* parasites collected in 25 countries in Asia, South America, Africa, and Oceania. Overall, we detected seven putative duplications located within 2 kb of the annotated *mdr1* gene (**Supplemental Fig. 2, Supplemental Table 5**), including a previously identified 35 kb duplication containing the entire *mdr1* gene [13]. In addition, we detected read pairs consistent with a deletion downstream of *mdr1* (and similar to the one we observed in our Cambodian isolates) in *P. vivax* isolates from Southeast Asia (Cambodia [122/169], Thailand [36/120], Vietnam [67/100], and Myanmar [3/8]), as well as in two of the 65 isolates from Colombia (**Supplemental Table 5**).

The identification of a similar deletion in two distant geographic locations raised the question of whether this deletion occurred once and spread from one region to the other or, alternatively, whether it derived from two independent events. To address this question, we examined the precise boundaries of the *mdr1* deletion in Southeast Asian and South American samples using whole genome sequence data from the Cambodian isolates and Colombian samples from MalariaGEN. We also included whole genome sequence data from the NIH-1993-F3 strain of *P. vivax* (a strain derived from the Salvador 1 strain initially isolated from a patient from El Salvador) [30]. The Colombian and NIH-1993-F3 samples appeared to carry the same deletion whose boundaries differed from all Cambodian samples and appeared to be smaller (**Supplemental Fig. 3**). To confirm this observation, we designed PCR primers surrounding the putative deletions (**Supplemental Table 2**) and amplified and sequenced DNA from Cambodian isolates carrying the deletion as well as from the NIH-1993-F3 clone. These analyses confirmed that the Cambodian *P. vivax* isolates carried the same 837 bp deletion compared to the P01 reference genome sequence, while NIH-1993-F3 carried a 487 bp deletion (**Supplemental Fig. 4**). Interestingly, both deletions shared one identical boundary, 25 bp after the 3’ end of the *mdr1* coding sequence, while their differing ends occurred at a similar sequence “TGTACA” (**Supplemental Fig. 4**). The different boundaries of the deleted sequences indicated that these two deletions most likely occurred independently (once in South America and once in Southeast Asia) and the observation of two independent deletions at the same locus suggests that they might have been driven by natural selection to alter an important functional element.

### The deletions downstream of mdr1 alter the mRNAs transcribed from the mdr1 gene and their expression level

**Figure 2:**
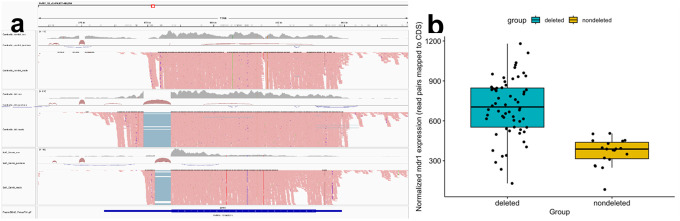
Effect of the downstream *mdr1* deletions on *mdr1* mRNA expression. **A)** Integrated Genome Viewer screenshot showing RNA-seq data mapping to the *mdr1*locus from the P01 reference genome: the tracks show data from, top to bottom, a Cambodian isolate without the deletion, a Cambodian sample with the deletion, and the Salvador 1 strain. **B)** Comparison of the normalized *mdr1* expression between *P. vivaxisolates* in which all clones carried the deletion (in blue) and isolates in which all clones have the non-deleted sequence (in yellow).

We next analyzed RNA-seq data generated from 95 Cambodian samples (**Supplemental Table 3**) to determine if the deletion had any consequence on *mdr1* mRNAs. While the deletion was located 25 bp after the end of the coding-sequence of *mdr1*, it led to a different mRNA being transcribed: in the parasites without the deletion, the 3’ UTR from *mdr1* is ~ 800 bp long, while it is ~ 1,300 bp long in parasites carrying the deletions and the nucleotide sequence is entirely different, except for the 25 bp immediately following the coding sequence (Fig. 2A). To examine if the South American deletion had a similar consequence on *mdr1* mRNA, we reanalyzed published RNA-seq data generated from Salvador I (the parental strain of NIH-1993-F3) [35] and observed a similar pattern: the DNA deletion led to the transcription of a different isoform of *mdr1* with a different 3’ UTR (Fig. 2A).

To determine if the deletion was associated with a change in the level of *mdr1* mRNA expression, we compared samples in which all *P. vivax* parasites carried the deletion (n = 61) to samples in which all parasites carried the non-deleted sequence (n = 19). After accounting for differences in mRNA length (see **Materials and Method** for details), we observed that *P. vivax* parasites carrying the deletion had a significantly greater expression of *mdr1* than parasites without the deletion (unpaired t-test, p = 9.75×10^− 9^) and displayed a nearly 2-fold increase in mRNA expression (Fig. 2B).

### The mdr1 3’UTR deletion is associated with differences in antimalarial drug susceptibility

**Figure 3:**
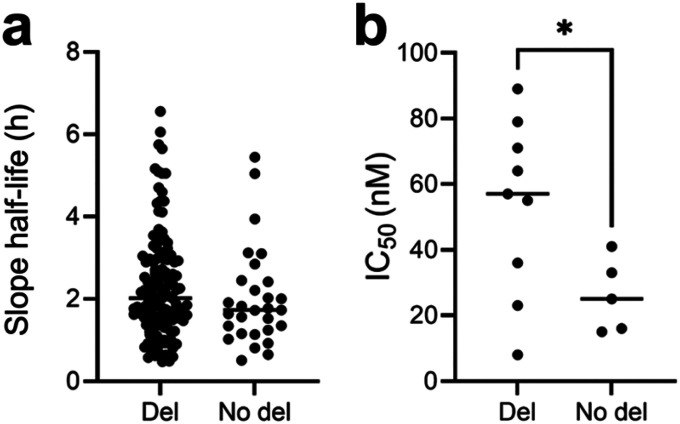
Association of *mdr1* deletion with antimalarial susceptibility. **A)** Distributions of slope half-life following *in vivo* artesunate treatment of patients infected with parasites with deletion and without deletion. **B)** Distributions of IC_50_ of parasites with deletion and without deletion when exposed to mefloquine *ex vivo*.

Since MDR1 has been associated with antimalarial drug resistance in *P. vivax* [12] and *P. falciparum* [14–16, 36], we then assessed whether the *mdr1* deletion was associated with reduced antimalarial susceptibility. First, we tested if the deletion was statistically associated with differences in parasite clearance upon artesunate treatment using estimates of the slope half-life measured directly from the patients studied here [22]. We compared 138 infections where all parasites carried the deletion, to 29 infections where all parasites carried the non-deleted sequences but did not observe any differences in slope half-life (p = 0.2311, Fig. 3A). Next, we genotyped the deletion in 14 *P. vivax* infections for which mefloquine susceptibility was measured *ex vivo*. Interestingly, parasites carrying the deletion had a significantly higher IC_50_ than parasites without the deletion (p = 0.0496, Fig. 3B).

### The mdr1 deletion increased in frequency among Cambodian P. vivax parasites since the introduction of mefloquine in ACTs

**Figure 4:**
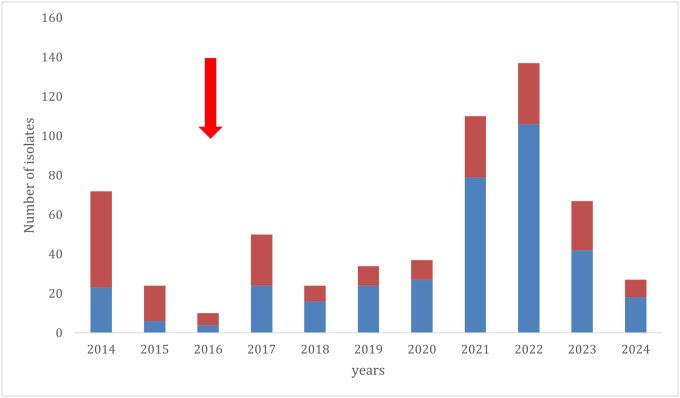
Longitudinal survey of the *mdr1*deletion in *P. vivax* isolates collected in Cambodia between 2014 and 2024. The blue stacks represent the numbers of *P. vivax* isolates carrying the deletion downstream of *mdr1* while the red stacks show the numbers of isolates without the deletion. The red arrow indicates the change in national treatment guidelines from DHA-PPQ to As-MQ.

Mefloquine has been used in ACT as the standard-of-care in Cambodia for treating uncomplicated vivax malaria since 2016–2017 [31]. To determine whether the change in the frontline antimalarial drug was associated with a change in the frequency of the *mdr1* deletion in the Cambodian *P. vivax* population, we screened a total of 592 isolates collected between 2014 and 2024. Overall, we observed a steady increase in the proportion of isolates carrying the deletion over time (p < 0.0001, Fig. 4), with ~ 30% of the parasites carrying the deletion before the introduction of mefloquine as standard-of-care compared to > 60% carrying the deletion today. Note that this pattern remained significant even if only parasites from eastern Cambodia are analyzed (**Supplemental Fig. 5**).

## Discussion

The multidrug resistance 1 gene in *Plasmodium* encodes an ATP-binding cassette transporter that is located on the membrane of the digestive vacuole where it regulates the flux of solutes through the membrane [37]. In *P. falciparum, mdr1* has been shown to modulate susceptibility to various antimalarial drugs through coding polymorphisms altering the amino acid sequences and copy number variations [14–16, 36]. Similarly in *P. vivax*, several studies have suggested that increased copy numbers of *mdr1* reduces antimalarial drug susceptibility [12, 13]. We showed here that *mdr1* is unusually affected by deletions and duplications in *P. vivax*. While we observed many sequence rearrangements among Cambodian *P. vivax* isolates, almost all of them occurred in large multigene families and the only common sequence rearrangement affecting a single copy protein-coding genes in 206 Cambodian isolates occurred immediately downstream of *mdr1*. This uniqueness of *mdr1* was further illustrated by a reanalysis of 592 *P. vivax* isolates from four continents that confirmed the presence of this deletion in Southeast Asian isolates but also revealed multiple, rarer, tandem duplications affecting this locus. Importantly, these analyses also revealed that a second deletion, overlapping but shorter, was present in some South American *P. vivax* isolates. The observation of two independent deletions affecting the same locus suggested that these deletions have functional consequences and may have been driven by positive selection.

In contrast to previous reports of *mdr1* polymorphisms, this common deletion observed in Cambodian isolates (as well as the one present in South American parasites) did not affect the coding region of *mdr1* but only modified its downstream DNA sequence. In addition, it was found on different genetic backgrounds and was linked to different *mdr1* amino acid sequences. RNA sequencing analyses confirmed that the deletion altered the gene expression of *mdr1* and produced transcripts with different 3’UTRs. While incompletely characterized in *Plasmodium*, 3’UTRs often play a critical role in mRNA stability in eukaryotes by binding RNAs and/or proteins that regulate mRNA stability and degradation [38]. Indeed, we showed that the presence of the deletion was associated with a two-fold increase in *mdr1* mRNA levels, suggesting that the deleted region, or the newly appended 3’ UTR sequence, contains important elements for mRNA regulation. Additional laboratory studies will be necessary to fully understand the molecular mechanisms responsible for these changes in expression and evaluate the consequences of these variable 3’ UTR sequences on transcription or mRNA stability and decay, as well as their impact on *mdr1* protein levels.

Similarly to the effect of increased *mdr1* copy number in *P. falciparum*, and to a lesser extent in *P. vivax*, we showed that the deletion downstream of *mdr1* was statistically associated with a higher IC_50_ to mefloquine. Moreover, our retrospective analyses of isolates collected in Cambodia since 2014 indicated that the frequency of the deletion increased after the introduction of mefloquine in ACT for uncomplicated vivax malaria in 2016–2018. Interestingly, we observed that the deletion was already present (although at lower frequency) in the oldest samples screened here, before the introduction of mefloquine in the standard-of-care for vivax malaria treatment. This observation, that is consistent with the lack of linkage disequilibrium between the deletion and the neighboring protein-coding sequence, could indicate that the deletion had been previously maintained in the *P. vivax* population, possibly due to “collateral” exposure of *P. vivax* parasites to mefloquine that has been extensively used against *P. falciparum* in Cambodia since 1983 [39, 40], or to advantages the *mdr1* deletion could confer against other antimalarial drugs [41–44].

Our observations, combined with the recent report of delayed clearance upon artemisinin treatment [22], suggest that *P. vivax* parasites could become less susceptible to Artesunate-Mefloquine therapy in Cambodia. These results mirror the patterns that were observed for *P. falciparum* (also in Cambodia) in the last decades that led to the emergence and spread of parasites carrying multiple resistance alleles and threatened malaria control efforts in the Greater Mekong region and worldwide. While we did not observe treatment failure associated with these putative multidrug resistance phenotypes in our Cambodian patient cohort, these findings are worrying and beg for intensified monitoring and control efforts to ensure that *P. vivax* parasites remain susceptible to current antimalarials and to limit the spread of potentially resistant parasites that could threaten ongoing efforts to eliminate vivax malaria from the Greater Mekong region.

## Data Availability

All sequence data generated in this study have been deposited in the National Center for Biotechnology Information (NCBI) Sequence Read Archive under the BioProject ID XXXX. Custom scripts are available at https://github.com/ko-katie.
